# Phase I/II study of ADXS11-001 or MEDI4736 immunotherapies alone and in combination, in patients with recurrent/metastatic cervical or human papillomavirus (HPV)-positive head and neck cancer

**DOI:** 10.1186/2051-1426-3-S2-P147

**Published:** 2015-11-04

**Authors:** Ezra EW Cohen, Kathleen N Moore, Brian M Slomovitz, Christine H Chung, Matthew L Anderson, Shannon R Morris, David Mauro, Barbara Burtness

**Affiliations:** 1 University of California, San Diego Moores Cancer Center, La Jolla, CA, USA; 2 Stephenson Oklahoma Cancer Center, Oklahoma City, OK, USA; 3 University of Miami, Sylvester Comprehensive Cancer Center, Miami, FL, USA; 4 Johns Hopkins University, Baltimore, MD, USA; 5 Baylor College of Medicine, Houston, TX, USA; 6 Medimmune, LLC, Gaithersburg, MD, USA; 7 Advaxis, Inc., Princeton, NJ, USA; 8 Yale University School of Medicine, New Haven, CT, USA

## Background

Approaches that target key HPV genes critical for cancer growth and metastasis may improve survival for individuals diagnosed with carcinomas of the uterine cervix or head and neck. ADXS11-001 is a live attenuated *Listeria monocytogenes (Lm)*-listeriolysin O (LLO) immunotherapy bioengineered to secrete an HPV-E7 tumor antigen as a truncated LLO-E7 fusion protein in cells capable of presenting antigen. This results in HPV-specific T-cell generation, reducing tumor protection in the tumor microenvironment. MEDI4736, an anti-programmed death-1 ligand (PD-L1) antibody, blocks the binding of PD-L1 to PD-1 and CD8, and relieves the inhibition of PD-L1–dependent immunosuppressive effects. Inhibition of PD-L1 binding increased the apparent immunologic potency/activity of ADXS11-001 in a preclinical study that showed the combination of ADXS11-011 and an anti–PD-L1 significantly retards tumor growth and prolongs survival in animals.

## Methods

This is an open-label, multicenter, 2-part, randomized Phase I/II study (NCT02291055). Patients (≥18 years) with squamous/nonsquamous cervical carcinoma or HPV-associated squamous cell cancer of the head and neck who progressed on ≥1 prior platinum-based therapy in the recurrent/metastatic setting are eligible. The primary objective of Phase I is to evaluate the safety and tolerability of ADXS11-001 plus MEDI4736 and select a recommended Phase II dose (RP2D) for the combination. The primary objective of Phase II is to evaluate the tumor response, progression-free survival (PFS), and safety of ADXS11-001 and MEDI4736 as monotherapy and in combination. Exploratory objectives for both phases will evaluate associations between biomarkers of immunologic response with tumor response and PFS. In Phase I, up to 18 patients will receive a fixed dose of ADXS11-001 (1×10^9^ colony-forming units [CFU]), while the dose of MEDI4736 will be escalated (starting at 3 mg/kg) according to a standard 3+3 design. In Phase II, patients (n≈48) will be randomized (1:1:2) to receive either ADXS11-001 (1×10^9^ CFU) or MEDI4736 (10 mg/kg) or both at the RP2D; all treatment arms will be stratified by disease. In both phases, ADXS11-001 will be administered every 4 weeks and MEDI4736 every 2 weeks. Patients will receive treatment up to 1 year or until they discontinue due to disease progression or unacceptable toxicity. Efficacy parameters will be evaluated by Response Evaluation Criteria in Solid Tumors (RECIST) and immune-related RECIST criteria, and safety determined using the Common Terminology Criteria for Adverse Events (CTCAE). Enrollment for this study is anticipated in July 2015.

## Trial registration

ClinicalTrials.gov identifier NCT02291055.

